# Crystal structure of di­chlorido­[2-(1*H*-imidazol-2-yl-κ*N* ^3^)imidazolato-κ*N*]bis­(tri-*n*-butyl­phosphane-κ*P*)rhodium(III)

**DOI:** 10.1107/S1600536814022454

**Published:** 2014-10-24

**Authors:** Jin-Long, Masahiro Ebihara

**Affiliations:** a Department of Chemistry and Biomolecular Science, Faculty of Engineering, Gifu University, Yanagido, Gifu 501-1193, Japan

**Keywords:** crystal structure, hydrogen bonding, bi­imidazole, rhodium complex

## Abstract

In the title compound, [Rh(C_6_H_5_N_4_)Cl_2_(C_12_H_27_P)_2_], the Rh^III^ ion is chelated by the singly deprotonated 2,2′-biimidazolate (Hbim^−^) ligand and coordinated by two chloride ions and two tri-*n*-butyl­phosphane ligands. The chloride ions and N atoms of the Hbim^−^ ligand lie in a plane where the sum of *X*—Rh—*X* angles between *cis* sites is 360°. The phosphane ligands occupy the sites perpendicular to the plane, completing the overall distorted octahedral coordination sphere. The complex forms a self-complementary hydrogen-bonded dimer with the inversion-related complex through N—H⋯N hydrogen bonds.

## Abstract 2

The Rh^III^ atom in the title compound shows a distorted octa­hedral coordination geometry. N—H⋯N hydrogen bonds involving the N atoms of the singly deprotonated biimidazolate ligands lead to the formation of inversion dimers.

## Chemical context   

Assembled structures and supra­molecules from metal complex modules have been one of the most actively investigated areas in coordination chemistry recently. The use of hydrogen bonding is a common method for the construction of structures. We have investigated dirhodium complexes with bi­imidazole (H_2_bim) or biimidazolate (Hbim^−^) ligands and two types of compounds [Rh_2_(H_2_bim)_4_ *L*_2_]^4+^ (*L* = H_2_O, MeOH, *etc*.) (Jin-Long *et al.*, 2014*a* ▶) and [Rh_2_(H_2_bim)_2_(O_2_C*R*)_2_(PPh_3_)_2_]^2+^ (*R* = propyl and butyl) (Jin-Long *et al.*, 2014*b* ▶) have been synthesized. We have tried to synthesize [Rh_2_(H_2_bim)_4_(P*R*_3_)_2_]^4+^, which is expected to have good solubility to organic solvents. However, the reaction of the dinuclear rhodium(II) complex [Rh_2_(H_2_bim)_4_(MeCN)_2_]^4+^ with PBu_3_ gave the mononuclear rhodium(III) compound [Rh(Hbim)Cl_2_(PBu_3_)_2_] (I). The source of the chloride ligands may be the chloro­form that was used as solvent.
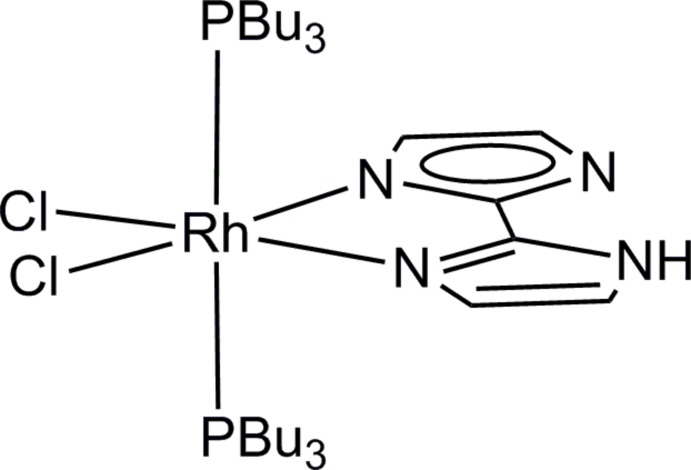


## Structural commentary   

**Figure 1:**
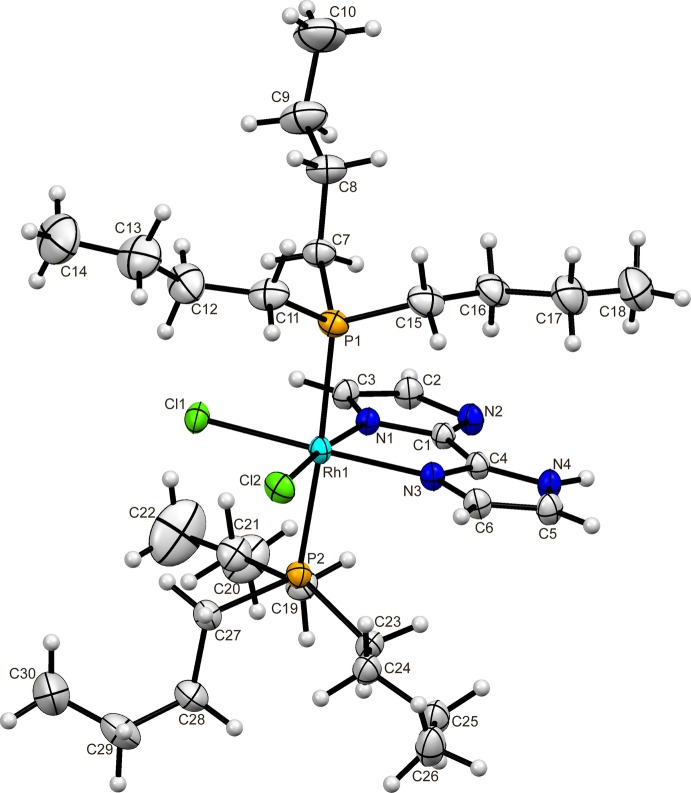
The mol­ecular structure of the title compound, showing the atom-labelling scheme. Displacement ellipsoids are drawn at the 30% probability level and H atoms are shown as small spheres of arbitrary radii.

In the structure of (I), the Rh^III^ ion is chelated by the singly deprotonated biimidazolate (Hbim^−^) ligand and coordinated by two chloride ions and two tri-*n*-butyl­phosphane ligands (Fig. 1 ▶). The chloride ions and N atoms of the Hbim^−^ ligand lie in a plane where the sum of *X*—Rh—*X* angles between *cis*-sites is 360°. The small bite angle of N1—Rh1—N3 [78.98 (7)°] makes the other angles wider than 90° [N1—Rh1—Cl1 93.28 (5), N3—Rh1—Cl2 94.18 (5) and Cl1—Rh1—Cl2 93.56 (2)°]. The phosphane ligands occupy the axial sites with a P1—Rh1—P2 angle of 176.29 (2)°.

## Supra­molecular features   

**Figure 2:**
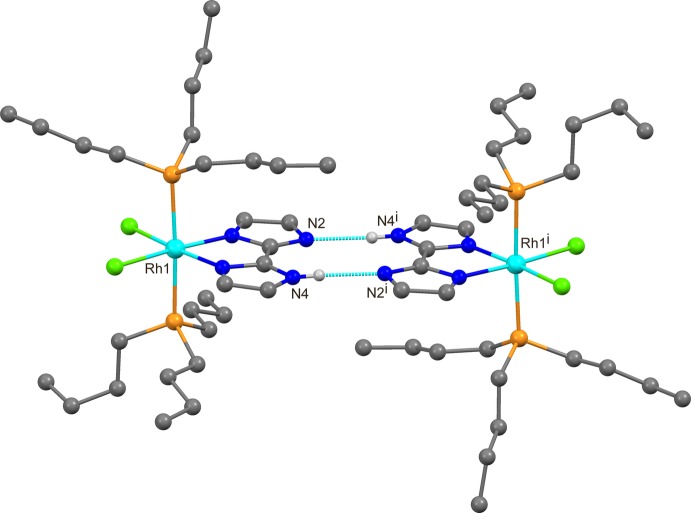
The hydrogen-bonded dimer structure of the title compound. H atoms except NH have been omitted for clarity. [Symmetry code: (i) −*x* + 1, −*y* + 1, −*z* + 1.]

Compound (I) is isostructural with the Re analogue [Re(Hbim)Cl_2_(PBu_3_)_2_] (Tadokoro *et al.*, 2007 ▶). The complex forms a self-complementary hydrogen-bonded dimer with the symmetry-related complex about the inversion centre at (½, ½, ½), as shown in Fig. 2 ▶. At is 2.772 (3) Å, the hydrogen-bonded N⋯N distance in the dimer is quite similar to those in [Re(Hbim)Cl_2_(PBu_3_)_2_] [2.771 (3) Å; Tadokoro *et al.*, 2007 ▶], [Re(Hbim)Cl_2_(PMe_3_)_2_] [2.775 (11) Å; Fortin *et al.*, 2001 ▶] and [Rh_2_(Hbim)_2_(O_2_C*R*)_2_(PPh_3_)_2_]_2_ [*R* = propyl: 2.774 (7), 2.737 (7), 2.735 (6) and 2.732 (7) Å, *R* = butyl: 2.752 (11) and 2.733 (12) Å; Jin-Long *et al.*, 2014*b* ▶].

## Database survey   

A search of the Cambridge Structural Database (Version 5.35, February 2014 update; Groom & Allen, 2014 ▶) reveals only twelve complexes that have an RhN_2_Cl_2_P_2_ core. Among them, two have a *cis*-NN, *cis*-ClCl and *trans*-PP geometry, *viz. cis*-di­chlorido-*trans-*bis­[(2-amino­eth­yl)di­phenyl­phosphino-*N*,*P*]rhodium chloride tetra­hydrate (Galsbøl *et al.*, 1986 ▶) and dichlorido-[2,2′-ethane-1,2-diylidenebis(1-phenyl­hydrazine)]bis­(tri­­phenyl­phosphane)rhodium triiodide (Patra *et al.*, 2011 ▶).

## Synthesis and crystallization   

[Rh_2_(H_2_bim)_4_(MeCN)_2_](BF_4_)_4_·H_2_O was prepared by a method described previously (Jin-Long *et al.*, 2014*b* ▶). A weighed amount of [Rh_2_(H_2_bim)_4_(MeCN)_2_](BF_4_)_4_·H_2_O (100 mg, 0.084 mmol) and tri­butyl­phosphane (0.21 ml, 0.840 mmol) in 5 ml of chloro­form was refluxed under an argon atmosphere for 30 min. From the resulting olive-green suspension, the solvent was removed by evaporation *in vacuo*. The olive-green solid changed to yellow when the flask was opened in air. The yellow solid was dissolved in MeOH and the insoluble solid was removed by filtration. Slow evap­oration of the solution gave yellow crystals of [Rh(Hbim)Cl_2_(PBu_3_)_2_] (56 mg, 93%). Analysis calculated for C_30_H_59_Cl_2_N_4_P_2_Rh: C 50.64, H 8.36, N 7.87%; found: C 50.37, H 8.37, N 8.05%.

## Refinement   

The hydrogen atom connected to the nitro­gen atom N4 was located by difference-Fourier methods and its positional and displacement parameters were refined. Other H atoms were placed in idealized positions and treated as riding atoms with C—H distances in the range 0.93–0.97 Å and *U*_iso_(H) = 1.2*U*_eq_(C) or 1.5*U*_eq_(C).

## Supplementary Material

Crystal structure: contains datablock(s) global, I. DOI: 10.1107/S1600536814022454/ff2131sup1.cif

Structure factors: contains datablock(s) I. DOI: 10.1107/S1600536814022454/ff2131Isup2.hkl

CCDC reference: 1028912

Additional supporting information:  crystallographic information; 3D view; checkCIF report

## Figures and Tables

**Table 1:** Selected bond lengths ()

Rh1N1	2.0322(18)	Rh1Cl2	2.3634(7)
Rh1N3	2.0538(19)	Rh1P2	2.3657(7)
Rh1Cl1	2.3450(7)	Rh1P1	2.3732(8)

**Table 2:** Hydrogen-bond geometry (, )

*D*H*A*	*D*H	H*A*	*D* *A*	*D*H*A*
N4H4N2^i^	0.80(3)	1.98(3)	2.772(3)	176(3)

Symmetry code: (i) 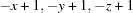.

**Table 3:** Experimental details

Crystal data	
Chemical formula	[Rh(C_6_H_5_N_4_)Cl_2_(C_12_H_27_P)_2_]
*M*_r_	711.56
Crystal system, space group	Monoclinic, *P*2_1_/*c*
Temperature (K)	296
*a*, *b*, *c* ()	11.969(2), 18.725(3), 16.894(3)
()	97.233(3)
*V* (^3^)	3756.1(11)
*Z*	4
Radiation type	Mo *K*
(mm^1^)	0.71
Crystal size (mm)	0.43 0.43 0.28

Data collection	
Diffractometer	Rigaku/MSC Mercury CCD
Absorption correction	Numerical (*NUMABS*; Higashi, 1999 ▶)
*T*_min_, *T*_max_	0.762, 0.871
No. of measured, independent and observed [*I* > 2(*I*)] reflections	28742, 8556, 7761
*R*_int_	0.025
(sin /)_max_ (^1^)	0.649

Refinement	
*R*[*F* ^2^ > 2(*F* ^2^)], *wR*(*F* ^2^), *S*	0.039, 0.100, 1.12
No. of reflections	8556
No. of parameters	362
H-atom treatment	H atoms treated by a mixture of independent and constrained refinement
_max_, _min_ (e ^3^)	1.12, 0.60

Computer programs: *CrystalClear* (Molecular Structure Corporation Rigaku, 2008 ▶), *SIR97* (Altomare *et al.*, 1999 ▶), *SHELXL97* (Sheldrick, 2008 ▶), *Mercury* (Macrae *et al.*, 2008 ▶) and *Yadokari-XG 2009* (Wakita, 2001 ▶; Kabuto *et al.*, 2009 ▶).

## supplementary crystallographic information

### Crystal data

**Table:**

[Rh(C_6_H_5_N_4_)Cl_2_(C_12_H_27_P)_2_]	*F*(000) = 1504
*M**_r_* = 711.56	*D*_x_ = 1.258 Mg m^−^^3^
Monoclinic, *P*2_1_/*c*	Mo *K*α radiation, λ = 0.71075 Å
Hall symbol: -P 2ybc	Cell parameters from 11010 reflections
*a* = 11.969 (2) Å	θ = 3.3–27.5°
*b* = 18.725 (3) Å	µ = 0.71 mm^−^^1^
*c* = 16.894 (3) Å	*T* = 296 K
β = 97.233 (3)°	Prism, yellow
*V* = 3756.1 (11) Å^3^	0.43 × 0.43 × 0.28 mm
*Z* = 4	

### Data collection

**Table:**

Rigaku/MSC Mercury CCD diffractometer	8556 independent reflections
Radiation source: Rotating Anode	7761 reflections with *I* > 2σ(*I*)
Graphite Monochromator monochromator	*R*_int_ = 0.025
Detector resolution: 14.6306 pixels mm^-1^	θ_max_ = 27.5°, θ_min_ = 3.4°
ω scans	*h* = −15→15
Absorption correction: numerical (*NUMABS*; Higashi, 1999)	*k* = −23→24
*T*_min_ = 0.762, *T*_max_ = 0.871	*l* = −21→13
28742 measured reflections	

### Refinement

**Table:**

Refinement on *F*^2^	Primary atom site location: structure-invariant direct methods
Least-squares matrix: full	Secondary atom site location: difference Fourier map
*R*[*F*^2^ > 2σ(*F*^2^)] = 0.039	Hydrogen site location: inferred from neighbouring sites
*wR*(*F*^2^) = 0.100	H atoms treated by a mixture of independent and constrained refinement
*S* = 1.12	*w* = 1/[σ^2^(*F*_o_^2^) + (0.0491*P*)^2^ + 1.6706*P*] where *P* = (*F*_o_^2^ + 2*F*_c_^2^)/3
8556 reflections	(Δ/σ)_max_ = 0.001
362 parameters	Δρ_max_ = 1.12 e Å^−^^3^
0 restraints	Δρ_min_ = −0.60 e Å^−^^3^

### Special details

**Table:**

Geometry. All e.s.d.'s (except the e.s.d. in the dihedral angle between two l.s. planes) are estimated using the full covariance matrix. The cell e.s.d.'s are taken into account individually in the estimation of e.s.d.'s in distances, angles and torsion angles; correlations between e.s.d.'s in cell parameters are only used when they are defined by crystal symmetry. An approximate (isotropic) treatment of cell e.s.d.'s is used for estimating e.s.d.'s involving l.s. planes.
Refinement. Refinement of *F*^2^ against ALL reflections. The weighted *R*-factor *wR* and goodness of fit *S* are based on *F*^2^, conventional *R*-factors *R* are based on *F*, with *F* set to zero for negative *F*^2^. The threshold expression of *F*^2^ > σ(*F*^2^) is used only for calculating *R*-factors(gt) *etc*. and is not relevant to the choice of reflections for refinement. *R*-factors based on *F*^2^ are statistically about twice as large as those based on *F*, and *R*- factors based on ALL data will be even larger.

### Fractional atomic coordinates and isotropic or equivalent isotropic displacement parameters (Å^2^)

**Table:**

	*x*	*y*	*z*	*U*_iso_*/*U*_eq_	
Rh1	0.402953 (14)	0.658731 (8)	0.730531 (9)	0.03314 (7)	
N1	0.46054 (16)	0.55992 (9)	0.70670 (10)	0.0351 (4)	
C1	0.47455 (19)	0.54765 (11)	0.62971 (12)	0.0342 (4)	
N2	0.51758 (18)	0.48329 (10)	0.61789 (11)	0.0420 (4)	
C2	0.5319 (2)	0.45319 (12)	0.69256 (14)	0.0453 (5)	
H2	0.5609	0.4078	0.7044	0.054*	
C3	0.4973 (2)	0.49954 (12)	0.74676 (14)	0.0420 (5)	
H3	0.4985	0.4914	0.8012	0.050*	
N3	0.40158 (17)	0.66435 (9)	0.60900 (11)	0.0362 (4)	
C4	0.44091 (19)	0.60582 (11)	0.57681 (12)	0.0354 (4)	
N4	0.43859 (19)	0.61395 (11)	0.49801 (11)	0.0415 (4)	
C5	0.3956 (2)	0.68098 (13)	0.47929 (14)	0.0452 (5)	
H5	0.3840	0.7012	0.4287	0.054*	
C6	0.3732 (2)	0.71191 (12)	0.54776 (14)	0.0420 (5)	
H6	0.3437	0.7574	0.5526	0.050*	
Cl1	0.41788 (5)	0.63754 (3)	0.86826 (3)	0.04604 (14)	
Cl2	0.33479 (6)	0.77629 (3)	0.74172 (4)	0.04849 (15)	
P1	0.21482 (6)	0.61643 (3)	0.72089 (4)	0.04473 (15)	
C7	0.2076 (2)	0.52534 (14)	0.75887 (17)	0.0509 (6)	
H7B	0.2536	0.4953	0.7292	0.061*	
H7A	0.2422	0.5253	0.8140	0.061*	
C8	0.0929 (3)	0.49015 (18)	0.7562 (2)	0.0681 (8)	
H8B	0.0428	0.5219	0.7803	0.082*	
H8A	0.0618	0.4829	0.7010	0.082*	
C9	0.0976 (4)	0.4194 (2)	0.7991 (3)	0.0923 (12)	
H9B	0.1572	0.3911	0.7809	0.111*	
H9A	0.1183	0.4282	0.8556	0.111*	
C10	−0.0076 (5)	0.3763 (3)	0.7890 (3)	0.1197 (18)	
H10C	−0.0655	0.4012	0.8123	0.180*	
H10A	0.0062	0.3310	0.8149	0.180*	
H10B	−0.0314	0.3689	0.7332	0.180*	
C11	0.1173 (3)	0.66831 (17)	0.7734 (2)	0.0648 (8)	
H11B	0.1228	0.7179	0.7577	0.078*	
H11A	0.0415	0.6525	0.7546	0.078*	
C12	0.1322 (3)	0.6655 (2)	0.8633 (2)	0.0830 (11)	
H12B	0.1286	0.6161	0.8801	0.100*	
H12A	0.2063	0.6836	0.8832	0.100*	
C13	0.0456 (4)	0.7075 (3)	0.8997 (3)	0.1032 (14)	
H13B	−0.0286	0.6925	0.8759	0.124*	
H13A	0.0540	0.7576	0.8871	0.124*	
C14	0.0531 (5)	0.6995 (3)	0.9884 (3)	0.128 (2)	
H14C	0.0302	0.6522	1.0011	0.192*	
H14A	0.0046	0.7338	1.0090	0.192*	
H14B	0.1294	0.7075	1.0120	0.192*	
C15	0.1371 (3)	0.61606 (17)	0.61953 (19)	0.0631 (8)	
H15B	0.0575	0.6122	0.6243	0.076*	
H15A	0.1487	0.6619	0.5951	0.076*	
C16	0.1666 (3)	0.55842 (18)	0.56344 (19)	0.0663 (8)	
H16B	0.1340	0.5137	0.5783	0.080*	
H16A	0.2478	0.5525	0.5698	0.080*	
C17	0.1262 (4)	0.5735 (2)	0.4762 (2)	0.0876 (12)	
H17B	0.0446	0.5765	0.4691	0.105*	
H17A	0.1553	0.6194	0.4620	0.105*	
C18	0.1617 (4)	0.5178 (3)	0.4204 (3)	0.1147 (17)	
H18C	0.2418	0.5113	0.4303	0.172*	
H18A	0.1412	0.5330	0.3663	0.172*	
H18B	0.1247	0.4734	0.4290	0.172*	
P2	0.59041 (5)	0.70149 (3)	0.74918 (4)	0.03851 (13)	
C19	0.6997 (2)	0.63231 (15)	0.76701 (16)	0.0509 (6)	
H19B	0.6833	0.5953	0.7271	0.061*	
H19A	0.7713	0.6535	0.7588	0.061*	
C20	0.7131 (3)	0.59719 (17)	0.84883 (18)	0.0597 (7)	
H20B	0.7373	0.6327	0.8891	0.072*	
H20A	0.6408	0.5787	0.8596	0.072*	
C21	0.7981 (4)	0.5368 (2)	0.8545 (3)	0.0958 (13)	
H21B	0.7713	0.5000	0.8163	0.115*	
H21A	0.8689	0.5547	0.8401	0.115*	
C22	0.8184 (5)	0.5042 (4)	0.9368 (4)	0.157 (3)	
H22C	0.8370	0.5412	0.9756	0.236*	
H22A	0.8795	0.4707	0.9390	0.236*	
H22B	0.7516	0.4799	0.9481	0.236*	
C23	0.6346 (2)	0.74769 (14)	0.66276 (15)	0.0498 (6)	
H23B	0.7160	0.7522	0.6709	0.060*	
H23A	0.6151	0.7180	0.6160	0.060*	
C24	0.5844 (3)	0.82146 (14)	0.64528 (15)	0.0519 (6)	
H24B	0.6139	0.8540	0.6875	0.062*	
H24A	0.5034	0.8190	0.6447	0.062*	
C25	0.6111 (3)	0.85031 (15)	0.56556 (17)	0.0597 (7)	
H25B	0.6920	0.8499	0.5649	0.072*	
H25A	0.5776	0.8193	0.5231	0.072*	
C26	0.5677 (4)	0.92541 (17)	0.55013 (18)	0.0744 (10)	
H26C	0.4904	0.9278	0.5597	0.112*	
H26A	0.5733	0.9382	0.4957	0.112*	
H26B	0.6118	0.9579	0.5852	0.112*	
C27	0.6197 (2)	0.76403 (14)	0.83187 (15)	0.0466 (5)	
H27B	0.5681	0.8040	0.8226	0.056*	
H27A	0.6039	0.7404	0.8803	0.056*	
C28	0.7397 (3)	0.79327 (19)	0.84555 (19)	0.0677 (8)	
H28B	0.7907	0.7542	0.8615	0.081*	
H28A	0.7589	0.8118	0.7954	0.081*	
C29	0.7582 (4)	0.8510 (2)	0.9074 (3)	0.0898 (12)	
H29B	0.8341	0.8695	0.9079	0.108*	
H29A	0.7064	0.8899	0.8920	0.108*	
C30	0.7435 (6)	0.8286 (4)	0.9867 (3)	0.145 (3)	
H30C	0.6670	0.8134	0.9878	0.217*	
H30A	0.7600	0.8677	1.0230	0.217*	
H30B	0.7935	0.7896	1.0024	0.217*	
H4	0.451 (3)	0.5844 (17)	0.4666 (19)	0.061 (9)*	

### Atomic displacement parameters (Å^2^)

**Table:**

	*U*^11^	*U*^22^	*U*^33^	*U*^12^	*U*^13^	*U*^23^
Rh1	0.03979 (11)	0.03010 (10)	0.02944 (10)	0.00293 (6)	0.00400 (7)	−0.00268 (6)
N1	0.0432 (10)	0.0324 (8)	0.0298 (8)	0.0030 (7)	0.0047 (7)	0.0000 (7)
C1	0.0420 (12)	0.0289 (9)	0.0316 (10)	0.0009 (8)	0.0045 (8)	−0.0015 (8)
N2	0.0581 (13)	0.0299 (9)	0.0379 (10)	0.0065 (8)	0.0059 (9)	−0.0025 (7)
C2	0.0613 (16)	0.0306 (10)	0.0434 (12)	0.0060 (10)	0.0036 (11)	0.0045 (9)
C3	0.0549 (14)	0.0347 (11)	0.0360 (11)	0.0032 (10)	0.0041 (10)	0.0067 (9)
N3	0.0468 (11)	0.0300 (9)	0.0312 (9)	0.0042 (7)	0.0025 (8)	0.0005 (7)
C4	0.0443 (12)	0.0297 (9)	0.0317 (10)	0.0016 (8)	0.0031 (9)	−0.0012 (8)
N4	0.0594 (13)	0.0364 (10)	0.0290 (9)	0.0034 (9)	0.0061 (8)	−0.0018 (8)
C5	0.0615 (16)	0.0381 (11)	0.0348 (11)	0.0037 (11)	0.0009 (10)	0.0058 (9)
C6	0.0555 (14)	0.0298 (10)	0.0394 (11)	0.0051 (10)	0.0003 (10)	0.0028 (9)
Cl1	0.0502 (3)	0.0575 (3)	0.0310 (3)	0.0025 (3)	0.0077 (2)	−0.0005 (2)
Cl2	0.0539 (4)	0.0342 (3)	0.0575 (4)	0.0056 (2)	0.0076 (3)	−0.0092 (2)
P1	0.0399 (3)	0.0415 (3)	0.0519 (4)	0.0004 (2)	0.0027 (3)	−0.0010 (3)
C7	0.0523 (15)	0.0420 (12)	0.0586 (15)	−0.0024 (11)	0.0080 (12)	0.0012 (11)
C8	0.0576 (19)	0.0624 (18)	0.086 (2)	−0.0136 (14)	0.0139 (16)	0.0066 (16)
C9	0.091 (3)	0.064 (2)	0.127 (3)	−0.0124 (19)	0.037 (3)	0.008 (2)
C10	0.128 (4)	0.087 (3)	0.149 (5)	−0.042 (3)	0.036 (4)	0.005 (3)
C11	0.0482 (16)	0.0554 (16)	0.094 (3)	0.0082 (12)	0.0207 (16)	−0.0015 (15)
C12	0.060 (2)	0.101 (3)	0.091 (3)	0.0125 (18)	0.0190 (19)	−0.026 (2)
C13	0.088 (3)	0.103 (3)	0.126 (4)	0.017 (2)	0.043 (3)	−0.022 (3)
C14	0.115 (4)	0.155 (5)	0.123 (4)	0.011 (4)	0.049 (3)	−0.040 (4)
C15	0.0487 (16)	0.0671 (18)	0.0690 (18)	−0.0015 (14)	−0.0100 (14)	0.0096 (15)
C16	0.0539 (18)	0.075 (2)	0.0646 (18)	−0.0078 (15)	−0.0114 (14)	−0.0020 (15)
C17	0.086 (3)	0.104 (3)	0.066 (2)	−0.016 (2)	−0.0176 (19)	0.006 (2)
C18	0.106 (4)	0.163 (5)	0.071 (2)	−0.029 (3)	−0.006 (2)	−0.021 (3)
P2	0.0404 (3)	0.0395 (3)	0.0361 (3)	0.0004 (2)	0.0067 (2)	−0.0019 (2)
C19	0.0420 (14)	0.0545 (15)	0.0571 (15)	0.0074 (11)	0.0100 (11)	−0.0025 (12)
C20	0.0512 (16)	0.0639 (17)	0.0629 (17)	0.0131 (13)	0.0025 (13)	0.0069 (14)
C21	0.081 (3)	0.100 (3)	0.109 (3)	0.048 (2)	0.019 (2)	0.029 (2)
C22	0.157 (6)	0.171 (6)	0.144 (5)	0.096 (5)	0.021 (4)	0.072 (4)
C23	0.0569 (16)	0.0526 (14)	0.0415 (13)	−0.0067 (12)	0.0131 (11)	0.0012 (11)
C24	0.0666 (18)	0.0475 (13)	0.0425 (13)	−0.0075 (12)	0.0104 (12)	0.0002 (11)
C25	0.082 (2)	0.0576 (16)	0.0400 (14)	−0.0052 (14)	0.0075 (14)	0.0011 (11)
C26	0.119 (3)	0.0578 (18)	0.0471 (16)	−0.0030 (18)	0.0148 (17)	0.0038 (13)
C27	0.0490 (14)	0.0469 (13)	0.0433 (13)	−0.0029 (11)	0.0031 (10)	−0.0054 (10)
C28	0.0605 (19)	0.080 (2)	0.0615 (18)	−0.0184 (16)	0.0024 (14)	−0.0104 (16)
C29	0.084 (3)	0.091 (3)	0.091 (3)	−0.030 (2)	−0.006 (2)	−0.020 (2)
C30	0.162 (6)	0.194 (6)	0.081 (3)	−0.081 (5)	0.025 (3)	−0.039 (4)

### Geometric parameters (Å, º)

**Table:**

Rh1—N1	2.0322 (18)	C15—H15B	0.9700
Rh1—N3	2.0538 (19)	C15—H15A	0.9700
Rh1—Cl1	2.3450 (7)	C16—C17	1.519 (5)
Rh1—Cl2	2.3634 (7)	C16—H16B	0.9700
Rh1—P2	2.3657 (7)	C16—H16A	0.9700
Rh1—P1	2.3732 (8)	C17—C18	1.502 (6)
N1—C1	1.352 (3)	C17—H17B	0.9700
N1—C3	1.362 (3)	C17—H17A	0.9700
C1—N2	1.335 (3)	C18—H18C	0.9600
C1—C4	1.434 (3)	C18—H18A	0.9600
N2—C2	1.373 (3)	C18—H18B	0.9600
C2—C3	1.363 (3)	P2—C27	1.823 (2)
C2—H2	0.9300	P2—C23	1.831 (2)
C3—H3	0.9300	P2—C19	1.839 (3)
N3—C4	1.335 (3)	C19—C20	1.521 (4)
N3—C6	1.375 (3)	C19—H19B	0.9700
C4—N4	1.337 (3)	C19—H19A	0.9700
N4—C5	1.378 (3)	C20—C21	1.516 (4)
N4—H4	0.80 (3)	C20—H20B	0.9700
C5—C6	1.350 (3)	C20—H20A	0.9700
C5—H5	0.9300	C21—C22	1.510 (6)
C6—H6	0.9300	C21—H21B	0.9700
P1—C7	1.828 (3)	C21—H21A	0.9700
P1—C11	1.831 (3)	C22—H22C	0.9600
P1—C15	1.843 (3)	C22—H22A	0.9600
C7—C8	1.518 (4)	C22—H22B	0.9600
C7—H7B	0.9700	C23—C24	1.521 (4)
C7—H7A	0.9700	C23—H23B	0.9700
C8—C9	1.507 (5)	C23—H23A	0.9700
C8—H8B	0.9700	C24—C25	1.522 (4)
C8—H8A	0.9700	C24—H24B	0.9700
C9—C10	1.487 (6)	C24—H24A	0.9700
C9—H9B	0.9700	C25—C26	1.511 (4)
C9—H9A	0.9700	C25—H25B	0.9700
C10—H10C	0.9600	C25—H25A	0.9700
C10—H10A	0.9600	C26—H26C	0.9600
C10—H10B	0.9600	C26—H26A	0.9600
C11—C12	1.507 (5)	C26—H26B	0.9600
C11—H11B	0.9700	C27—C28	1.527 (4)
C11—H11A	0.9700	C27—H27B	0.9700
C12—C13	1.494 (5)	C27—H27A	0.9700
C12—H12B	0.9700	C28—C29	1.500 (5)
C12—H12A	0.9700	C28—H28B	0.9700
C13—C14	1.498 (7)	C28—H28A	0.9700
C13—H13B	0.9700	C29—C30	1.437 (6)
C13—H13A	0.9700	C29—H29B	0.9700
C14—H14C	0.9600	C29—H29A	0.9700
C14—H14A	0.9600	C30—H30C	0.9600
C14—H14B	0.9600	C30—H30A	0.9600
C15—C16	1.507 (5)	C30—H30B	0.9600
			
N1—Rh1—N3	78.98 (7)	C16—C15—H15A	108.0
N1—Rh1—Cl1	93.28 (5)	P1—C15—H15A	108.0
N3—Rh1—Cl1	172.15 (5)	H15B—C15—H15A	107.3
N1—Rh1—Cl2	173.16 (5)	C15—C16—C17	114.0 (3)
N3—Rh1—Cl2	94.18 (5)	C15—C16—H16B	108.8
Cl1—Rh1—Cl2	93.56 (2)	C17—C16—H16B	108.8
N1—Rh1—P2	89.71 (6)	C15—C16—H16A	108.8
N3—Rh1—P2	90.22 (6)	C17—C16—H16A	108.8
Cl1—Rh1—P2	88.43 (2)	H16B—C16—H16A	107.7
Cl2—Rh1—P2	90.30 (2)	C18—C17—C16	113.5 (4)
N1—Rh1—P1	91.44 (6)	C18—C17—H17B	108.9
N3—Rh1—P1	93.46 (6)	C16—C17—H17B	108.9
Cl1—Rh1—P1	87.98 (2)	C18—C17—H17A	108.9
Cl2—Rh1—P1	88.98 (2)	C16—C17—H17A	108.9
P2—Rh1—P1	176.29 (2)	H17B—C17—H17A	107.7
C1—N1—C3	105.41 (18)	C17—C18—H18C	109.5
C1—N1—Rh1	115.57 (14)	C17—C18—H18A	109.5
C3—N1—Rh1	138.91 (15)	H18C—C18—H18A	109.5
N2—C1—N1	113.22 (18)	C17—C18—H18B	109.5
N2—C1—C4	132.48 (19)	H18C—C18—H18B	109.5
N1—C1—C4	114.30 (18)	H18A—C18—H18B	109.5
C1—N2—C2	103.81 (18)	C27—P2—C23	105.10 (12)
C3—C2—N2	110.0 (2)	C27—P2—C19	105.24 (13)
C3—C2—H2	125.0	C23—P2—C19	101.09 (13)
N2—C2—H2	125.0	C27—P2—Rh1	114.03 (9)
N1—C3—C2	107.52 (19)	C23—P2—Rh1	114.71 (9)
N1—C3—H3	126.2	C19—P2—Rh1	115.25 (10)
C2—C3—H3	126.2	C20—C19—P2	116.45 (19)
C4—N3—C6	106.95 (18)	C20—C19—H19B	108.2
C4—N3—Rh1	113.98 (14)	P2—C19—H19B	108.2
C6—N3—Rh1	139.07 (15)	C20—C19—H19A	108.2
N3—C4—N4	110.37 (19)	P2—C19—H19A	108.2
N3—C4—C1	117.07 (18)	H19B—C19—H19A	107.3
N4—C4—C1	132.5 (2)	C21—C20—C19	111.9 (3)
C4—N4—C5	107.01 (19)	C21—C20—H20B	109.2
C4—N4—H4	127 (2)	C19—C20—H20B	109.2
C5—N4—H4	125 (2)	C21—C20—H20A	109.2
C6—C5—N4	107.6 (2)	C19—C20—H20A	109.2
C6—C5—H5	126.2	H20B—C20—H20A	107.9
N4—C5—H5	126.2	C22—C21—C20	112.8 (4)
C5—C6—N3	108.1 (2)	C22—C21—H21B	109.0
C5—C6—H6	126.0	C20—C21—H21B	109.0
N3—C6—H6	126.0	C22—C21—H21A	109.0
C7—P1—C11	105.28 (14)	C20—C21—H21A	109.0
C7—P1—C15	106.38 (14)	H21B—C21—H21A	107.8
C11—P1—C15	100.06 (16)	C21—C22—H22C	109.5
C7—P1—Rh1	111.95 (9)	C21—C22—H22A	109.5
C11—P1—Rh1	116.53 (11)	H22C—C22—H22A	109.5
C15—P1—Rh1	115.36 (11)	C21—C22—H22B	109.5
C8—C7—P1	118.5 (2)	H22C—C22—H22B	109.5
C8—C7—H7B	107.7	H22A—C22—H22B	109.5
P1—C7—H7B	107.7	C24—C23—P2	115.92 (18)
C8—C7—H7A	107.7	C24—C23—H23B	108.3
P1—C7—H7A	107.7	P2—C23—H23B	108.3
H7B—C7—H7A	107.1	C24—C23—H23A	108.3
C9—C8—C7	112.9 (3)	P2—C23—H23A	108.3
C9—C8—H8B	109.0	H23B—C23—H23A	107.4
C7—C8—H8B	109.0	C23—C24—C25	111.9 (2)
C9—C8—H8A	109.0	C23—C24—H24B	109.2
C7—C8—H8A	109.0	C25—C24—H24B	109.2
H8B—C8—H8A	107.8	C23—C24—H24A	109.2
C10—C9—C8	116.2 (4)	C25—C24—H24A	109.2
C10—C9—H9B	108.2	H24B—C24—H24A	107.9
C8—C9—H9B	108.2	C26—C25—C24	112.2 (3)
C10—C9—H9A	108.2	C26—C25—H25B	109.2
C8—C9—H9A	108.2	C24—C25—H25B	109.2
H9B—C9—H9A	107.4	C26—C25—H25A	109.2
C9—C10—H10C	109.5	C24—C25—H25A	109.2
C9—C10—H10A	109.5	H25B—C25—H25A	107.9
H10C—C10—H10A	109.5	C25—C26—H26C	109.5
C9—C10—H10B	109.5	C25—C26—H26A	109.5
H10C—C10—H10B	109.5	H26C—C26—H26A	109.5
H10A—C10—H10B	109.5	C25—C26—H26B	109.5
C12—C11—P1	117.9 (2)	H26C—C26—H26B	109.5
C12—C11—H11B	107.8	H26A—C26—H26B	109.5
P1—C11—H11B	107.8	C28—C27—P2	115.66 (19)
C12—C11—H11A	107.8	C28—C27—H27B	108.4
P1—C11—H11A	107.8	P2—C27—H27B	108.4
H11B—C11—H11A	107.2	C28—C27—H27A	108.4
C13—C12—C11	113.4 (4)	P2—C27—H27A	108.4
C13—C12—H12B	108.9	H27B—C27—H27A	107.4
C11—C12—H12B	108.9	C29—C28—C27	114.7 (3)
C13—C12—H12A	108.9	C29—C28—H28B	108.6
C11—C12—H12A	108.9	C27—C28—H28B	108.6
H12B—C12—H12A	107.7	C29—C28—H28A	108.6
C12—C13—C14	113.7 (4)	C27—C28—H28A	108.6
C12—C13—H13B	108.8	H28B—C28—H28A	107.6
C14—C13—H13B	108.8	C30—C29—C28	114.4 (4)
C12—C13—H13A	108.8	C30—C29—H29B	108.7
C14—C13—H13A	108.8	C28—C29—H29B	108.7
H13B—C13—H13A	107.7	C30—C29—H29A	108.7
C13—C14—H14C	109.5	C28—C29—H29A	108.7
C13—C14—H14A	109.5	H29B—C29—H29A	107.6
H14C—C14—H14A	109.5	C29—C30—H30C	109.5
C13—C14—H14B	109.5	C29—C30—H30A	109.5
H14C—C14—H14B	109.5	H30C—C30—H30A	109.5
H14A—C14—H14B	109.5	C29—C30—H30B	109.5
C16—C15—P1	117.1 (2)	H30C—C30—H30B	109.5
C16—C15—H15B	108.0	H30A—C30—H30B	109.5
P1—C15—H15B	108.0		
			
N3—Rh1—N1—C1	2.99 (16)	Cl2—Rh1—P1—C11	−30.31 (12)
Cl1—Rh1—N1—C1	−175.70 (16)	N1—Rh1—P1—C15	−86.56 (13)
P2—Rh1—N1—C1	−87.29 (16)	N3—Rh1—P1—C15	−7.52 (13)
P1—Rh1—N1—C1	96.24 (16)	Cl1—Rh1—P1—C15	−179.79 (12)
N3—Rh1—N1—C3	178.3 (3)	Cl2—Rh1—P1—C15	86.61 (12)
Cl1—Rh1—N1—C3	−0.4 (3)	C11—P1—C7—C8	53.0 (3)
P2—Rh1—N1—C3	88.0 (3)	C15—P1—C7—C8	−52.6 (3)
P1—Rh1—N1—C3	−88.5 (3)	Rh1—P1—C7—C8	−179.5 (2)
C3—N1—C1—N2	0.2 (3)	P1—C7—C8—C9	−172.4 (3)
Rh1—N1—C1—N2	176.96 (16)	C7—C8—C9—C10	−171.0 (4)
C3—N1—C1—C4	−179.7 (2)	C7—P1—C11—C12	53.7 (3)
Rh1—N1—C1—C4	−2.9 (3)	C15—P1—C11—C12	163.9 (3)
N1—C1—N2—C2	−0.2 (3)	Rh1—P1—C11—C12	−71.0 (3)
C4—C1—N2—C2	179.6 (3)	P1—C11—C12—C13	−177.9 (3)
C1—N2—C2—C3	0.1 (3)	C11—C12—C13—C14	174.6 (4)
C1—N1—C3—C2	−0.1 (3)	C7—P1—C15—C16	−49.8 (3)
Rh1—N1—C3—C2	−175.67 (19)	C11—P1—C15—C16	−159.2 (3)
N2—C2—C3—N1	0.0 (3)	Rh1—P1—C15—C16	75.0 (3)
N1—Rh1—N3—C4	−2.58 (16)	P1—C15—C16—C17	−163.0 (3)
Cl2—Rh1—N3—C4	177.40 (16)	C15—C16—C17—C18	176.7 (3)
P2—Rh1—N3—C4	87.08 (16)	N1—Rh1—P2—C27	−140.17 (11)
P1—Rh1—N3—C4	−93.37 (16)	N3—Rh1—P2—C27	140.85 (11)
N1—Rh1—N3—C6	178.7 (3)	Cl1—Rh1—P2—C27	−46.89 (10)
Cl2—Rh1—N3—C6	−1.3 (3)	Cl2—Rh1—P2—C27	46.67 (10)
P2—Rh1—N3—C6	−91.6 (3)	N1—Rh1—P2—C23	98.54 (11)
P1—Rh1—N3—C6	87.9 (3)	N3—Rh1—P2—C23	19.57 (11)
C6—N3—C4—N4	−0.1 (3)	Cl1—Rh1—P2—C23	−168.17 (10)
Rh1—N3—C4—N4	−179.23 (16)	Cl2—Rh1—P2—C23	−74.61 (10)
C6—N3—C4—C1	−179.0 (2)	N1—Rh1—P2—C19	−18.28 (11)
Rh1—N3—C4—C1	1.9 (3)	N3—Rh1—P2—C19	−97.26 (11)
N2—C1—C4—N3	−179.2 (2)	Cl1—Rh1—P2—C19	75.01 (10)
N1—C1—C4—N3	0.7 (3)	Cl2—Rh1—P2—C19	168.56 (10)
N2—C1—C4—N4	2.2 (5)	C27—P2—C19—C20	52.7 (2)
N1—C1—C4—N4	−178.0 (2)	C23—P2—C19—C20	161.8 (2)
N3—C4—N4—C5	0.0 (3)	Rh1—P2—C19—C20	−73.9 (2)
C1—C4—N4—C5	178.6 (3)	P2—C19—C20—C21	175.0 (3)
C4—N4—C5—C6	0.2 (3)	C19—C20—C21—C22	176.3 (4)
N4—C5—C6—N3	−0.3 (3)	C27—P2—C23—C24	−53.6 (2)
C4—N3—C6—C5	0.2 (3)	C19—P2—C23—C24	−162.9 (2)
Rh1—N3—C6—C5	179.00 (19)	Rh1—P2—C23—C24	72.5 (2)
N1—Rh1—P1—C7	35.28 (11)	P2—C23—C24—C25	−170.9 (2)
N3—Rh1—P1—C7	114.32 (11)	C23—C24—C25—C26	−176.5 (3)
Cl1—Rh1—P1—C7	−57.95 (10)	C23—P2—C27—C28	−53.1 (3)
Cl2—Rh1—P1—C7	−151.55 (10)	C19—P2—C27—C28	53.1 (2)
N1—Rh1—P1—C11	156.52 (13)	Rh1—P2—C27—C28	−179.6 (2)
N3—Rh1—P1—C11	−124.43 (13)	P2—C27—C28—C29	173.0 (3)
Cl1—Rh1—P1—C11	63.29 (12)	C27—C28—C29—C30	64.2 (5)

### Hydrogen-bond geometry (Å, º)

**Table:**

*D*—H···*A*	*D*—H	H···*A*	*D*···*A*	*D*—H···*A*
N4—H4···N2^i^	0.80 (3)	1.98 (3)	2.772 (3)	176 (3)

Symmetry code: (i) −*x*+1, −*y*+1, −*z*+1.
